# Review of "The Blood-Cerebrospinal Fluid Barrier" by Wei Zheng and Adam Chodobski (editors)

**DOI:** 10.1186/1743-8454-3-12

**Published:** 2006-12-08

**Authors:** Hazel C Jones

**Affiliations:** 1 Department of Pharmacology, University of Florida, Gainesville, FL 32610, USA; 2 Gagle Brook House, Chesterton, Bicester, Oxon OX26 1UF, UK

## Abstract

This multi-author volume on the blood-cerebrospinal fluid barrier summarizes past and current research in the areas of choroid plexus and cerebrospinal fluid in health and disease.

## 

The blood-cerebrospinal fluid barrier functions together with the blood-brain barrier and the meninges, to control the internal environment of the brain. Sited at the choroid plexus epithelium, it secretes the cerebrospinal fluid (CSF), which circulates through the ventricles and around the outside of the brain and spinal cord. Originally assumed to pass an ultra filtrate of plasma into the brain ventricles, the choroid plexus was subsequently shown to secrete a homeostatically controlled electrolyte solution, the CSF [1]. In the last twenty years the choroidal epithelium has emerged as a complex organ with many additional functions that include neuroendocrine signaling, neuroimmune and neuroinflammatory responses, drug and toxin metabolism and transport. There is an increasing awareness that improved knowledge of the role of the choroid plexus in brain function has the potential to open new avenues for the treatment and prevention of neurological disorders.

There has been a requirement for a comprehensive review on the choroid plexus and blood-CSF barrier for a number of years [2]. This multi-author book edited by Wei Zheng and Adam Chodobski has 24 chapters and 51 contributors. The chapters have a consistent style and each one has a complete reference list. The book fulfills an important need for research scientists in the field of choroid plexus and CSF and as a reference book for neuroscientists, clinicians and toxicologists. Section 1 is devoted to *choroid plexus development and morphology*, section 2 to *barrier physiology and molecular biology*, section 3 to the *barrier in disease states *and section 4 to *in vivo and in vitro models of the barrier*. Many topics are covered in this book and it is possible to select only a few for mention as being of particular interest. The proteins and lipids that make up the intercellular tight junctions are being elucidated at the molecular level, but how permeability is regulated awaits further investigation. The physiological regulation of CSF secretion is another important topic that has received much attention and is yet to be fully elucidated, together with the expression and role of ion transporters and channels. Knowledge of transporters for toxic substances and foreign chemicals and their metabolism by the choroid plexus is expanding rapidly, as are the fundamentals of nucleoside and metal transport and the role of choroid plexus peptides in hormonal signaling in the brain. Choroid plexus function changes with disease and aging and knowledge of the processes involved is important for understanding CNS disease states, such as Alzheimer's, Parkinson's, HIV, and disorders of CSF circulation. The final section provides the reader with a detailed review of the techniques used for investigation of choroid plexus function including *in situ *perfusion, cell culture and whole animal methods.

In conclusion, 'The Blood-Cerebrospinal Fluid Barrier' is a comprehensive book that covers all aspects of the subject and should be on the bookshelf of all researchers involved in brain barriers and brain homeostasis.

## Competing interests

The author(s) declare that they have no competing interests.

## Authors' contributions

Sole author
